# Meninges harbor cells expressing neural precursor markers during development and adulthood

**DOI:** 10.3389/fncel.2015.00383

**Published:** 2015-10-02

**Authors:** Francesco Bifari, Valeria Berton, Annachiara Pino, Marijana Kusalo, Giorgio Malpeli, Marzia Di Chio, Emanuela Bersan, Eliana Amato, Aldo Scarpa, Mauro Krampera, Guido Fumagalli, Ilaria Decimo

**Affiliations:** ^1^Section of Hematology, Stem Cell Research Laboratory, Department of Medicine, University of VeronaVerona, Italy; ^2^Section of Pharmacology, Department of Diagnostics and Public Health, University of VeronaVerona, Italy; ^3^Section of Pathological Anatomy, Department of Diagnostics and Public Health, University of VeronaVerona, Italy

**Keywords:** meninges, neural precursor cells, fractones, nestin, brain development, proliferation, neural stem cell niche

## Abstract

Brain and skull developments are tightly synchronized, allowing the cranial bones to dynamically adapt to the brain shape. At the brain-skull interface, meninges produce the trophic signals necessary for normal corticogenesis and bone development. Meninges harbor different cell populations, including cells forming the endosteum of the cranial vault. Recently, we and other groups have described the presence in meninges of a cell population endowed with neural differentiation potential *in vitro* and, after transplantation, *in vivo*. However, whether meninges may be a niche for neural progenitor cells during embryonic development and in adulthood remains to be determined. In this work we provide the first description of the distribution of neural precursor markers in rat meninges during development up to adulthood. We conclude that meninges share common properties with the classical neural stem cell niche, as they: (*i*) are a highly proliferating tissue; (*ii*) host cells expressing neural precursor markers such as nestin, vimentin, Sox2 and doublecortin; and (*iii*) are enriched in extracellular matrix components (e.g., fractones) known to bind and concentrate growth factors. This study underlines the importance of meninges as a potential niche for endogenous precursor cells during development and in adulthood.

## Introduction

Over the last years, new and unexpected roles for meninges have emerged (Decimo et al., 2012a; Richtsmeier and Flaherty, 2013; Bjornsson et al., 2015). Not just a protective fluid-filled membranous sac enclosing the brain, meninges form a complex microenvironment endowed with soluble trophic factors, extracellular matrices and cells playing fundamental roles in both skull and brain development (Richtsmeier and Flaherty, 2013; Bjornsson et al., 2015). In the developing rat, meninges begin to form at embryonic day 11 (E11) appearing as an undifferentiated mesenchymal network of cells located between the epidermis and the brain (Angelov and Vasilev, 1989). The bones of the skull start to form by E14, whereas meninges complete their differentiation and appear as a three-layered tissue (the outer dura mater, the inner pia mater and the intermediate arachnoid) only after E19 (Mercier and Hatton, 2000; Mercier et al., 2002; Kokovay et al., 2008; Bjornsson et al., 2015). At the end of brain development, meninges penetration and distribution inside the central nervous system (CNS) parenchyma is abundant and complex (Mercier and Hatton, 2000; Mercier et al., 2002). Indeed, extroflexions of the pia and arachnoid membranes (leptomeninges) form a perivascular space (Virchow–Robin space) around every vessel of the CNS (Reina-De La Torre et al., 1998; Rodriguez-Baeza et al., 1998).

Meninges-derived extracellular matrix components (e.g., laminin, heparan sulfate proteoglycans, collagen IV and fibronectin) have been shown to be essential for the correct development of the cortex (Halfter et al., 2002; Beggs et al., 2003). In addition, several molecules playing critical functions in cranial bone and brain development and homeostasis have been shown to be produced by meninges (Radakovits et al., 2009; Richtsmeier and Flaherty, 2013); these include fibroblast growth factors (FGFs) (Mercier and Hatton, 2001), insulin-like growth factor-II (Stylianopoulou et al., 1988), retinoic acid (RA) (Siegenthaler et al., 2009), stromal cell-derived factor-1 (SDF-1, also referred to as CXCL12) (Borrell and Marin, 2006; Belmadani et al., 2015) and transforming growth factor beta family proteins (Choe et al., 2014).

Meningeal cells of the dura mater may function as endosteum of the cranial vault (Adeeb et al., 2012; Richtsmeier and Flaherty, 2013). Moreover, we have recently found that leptomeninges of adult rodent brain and spinal cord host a population of cells expressing the neural precursor markers nestin and doublecortin (DCX) (Bifari et al., 2009; Decimo et al., 2011). A similar cell population was also described in human meninges (Decimo et al., 2011; Petricevic et al., 2011). Cells isolated from both brain and spinal cord leptomeninges could be differentiated into neurons and oligodendrocytes *in vitro*; after transplantation *in vivo* these cells integrate in hippocampal CA1 region acquiring neuronal morphology (Bifari et al., 2009). Of note, following injury meningeal cells increase their proliferation rate, migrate into the parenchyma, contribute to the injury-induced reaction (Decimo et al., 2011; Kumar et al., 2014) and increase their expression of neural progenitor markers (Decimo et al., 2011; Nakagomi et al., 2011, 2012; Ninomiya et al., 2013).

Interestingly, this pattern of reactivity to injury (increased proliferation, expression of progenitor markers and migration) is a typical feature of the well-described neural stem cell niche of the subventricular zone (SVZ) (Decimo et al., 2012b; Bjornsson et al., 2015). Here, the niche shows a peculiar microenvironment that provides conditions for maintenance of the stem cell pools in a quiescent state as well as signals for activation and differentiation when neurogenesis is required (Scadden, 2006; Decimo et al., 2012a,b; Bjornsson et al., 2015).

Considering the fundamental role of meningeal cells during brain development, the presence of cells expressing markers of stemness and their activation following CNS injury, we asked whether leptomeninges share some of the features of a neural stem cell niche. To this aim we analyzed by morphological, molecular and biochemical criteria: (i) the number and the proliferation rate of leptomeningeal cells; (ii) the presence and the distribution of cells expressing neural progenitor markers; and (iii) the distribution of some of the known extracellular components of neural niches. Since the primary feature of a stem cell niche is the capability to harbor and maintain precursors, in this study we analyzed rat brain leptomeninges in embryo, at birth, during weaning and in adult animals.

## Materials and methods

### Tissue preparation for immunofluorescence

Animal housing and all the protocols involving the use of experimental animals in this study were carried out in accordance with the recommendations of the Italian Ministry of Health (approved protocol N. 154/2014-B). Sprague-Dawley (SD) rats at different developmental stages (embryonic day 14: E14; embryonic day 20: E20; at birth: P0; after weaning at postnatal day 15: P15; young adult at 6–8 weeks and mature adult at 24 weeks) were anesthetized by intraperitoneal injection with chloral hydrate (350 mg/kg) and sacrificed by intracardial perfusion of PBS with 4% paraformaldehyde (PFA)/4% sucrose (pH 7.4) solution. Brains were extracted, fixed in 4% PFA solution and transferred into 10% and subsequently 30% sucrose solution. By cryostat cutting, 40 μm thick coronal brain sections were obtained and processed by immunofluorescence.

### Immunofluorescence and quantitative analysis

Brain slices were incubated for 2 h in blocking solution (5%FBS/3%BSA/0.3% Triton X-100 in PBS) and then incubated overnight at 4°C with primary antibodies. Primary antibodies were detected with appropriate secondary antibodies for 4 h at 4°C in blocking solution. Slices were incubated for 10 min with the nuclear dye TO-PRO 3 (Invitrogen). Staining for the nuclear marker of proliferation Ki67 required antigen retrieval prior to the standard protocol applied in this study; slides were therefore incubated for 30 s in citrate buffer (10 mM trisodium citrate dihydrate/0.05% Tween-20 pH 6.0). Quantification of Ki67-, nestin-, vimentin-, Sox2-, and DCX-positive cells and nuclei was done by counting positive cells above the basal lamina (identified by laminin reactivity) in at least 18 sections for each time point (*n* ≥ 3 animals analyzed). Acquisition parameter settings (pinhole, gain, offset, laser intensity) were kept fixed for each channel in different sessions of observation at the confocal microscope.

### Antibodies

The following primary antibodies were used: anti-nestin (mouse, 1:1000, BD Pharmingen), anti-laminin (rabbit, 1:1000, Sigma), anti-Ki67 (rabbit, 1:100, Abcam), anti-vimentin (chicken, 1:1000, Millipore), anti-Sox2 (goat, 1:200, Santa Cruz), anti-DCX (goat, 1:100, Santa Cruz), anti-Tuj1 (mouse, 1:1000, Covance) and anti-heparan sulfate (mouse, 1:500, US Biological).

The following secondary antibodies were used: goat anti-mouse CY3 (Amersham), donkey anti-mouse 488 (Molecular Probes), goat anti-rabbit 488 (Molecular Probes), donkey anti-rabbit 488 (Molecular Probes), rabbit anti-chicken CY3 (Chemicon), donkey anti-goat 546 (Molecular Probe). Nuclei were stained with the nuclear marker TO-PRO3 (Invitrogen).

### Laser capture microdissection

Frozen sections of rat brains (13 μm thick) at each stage of development (E20, P0, P15, and 6–8 weeks adult) were cut on Cryostat CM1950 (Leica Microsystems) and mounted on PEN-membrane coated glass slides (Leica Microsystems). After fixation in 70% ethanol and staining with hematoxylin, 1000 cells from meninges and 6–8 weeks adult SVZ were dissected with LMD6000 instrument (Leica Microsystems). Cells were collected in the cap of 0.5 ml tube containing the lysis buffer from Picopure RNA Isolation kit (Arcturus) and RNA extraction was performed according to manufacturer's protocol. First strand cDNA was synthesized with random primers using SuperScript II Reverse Transcriptase (Invitrogen) and used for subsequent qRT-PCR analysis.

### Quantitative RT (reverse transcription)–PCR analysis (qRT-PCR)

Total RNA was purified with Trizol reagent (Invitrogen) and retrotranscribed to cDNA by reverse transcriptase AMV contained in the First Strand cDNA Synthesis Kit (Roche). qRT-PCR reactions were carried out in 20 μl total volume containing 10 ng of cDNA (RNA equivalent), 1 μl Power SYBR Green I Master Mix or Taqman Universal PCR Master Mix (Applied Biosystems), 0.4 μM primers forward and reverse or 1/20 Taqman probe. After a starting denaturation for 10 min at 95°C, 40 PCR cycles (15 s 95°C and 1 min 60°C) were carried out on ABI PRISM 7900HT SDS instrument (Applied Biosystems).

Forward and reverse 5_−3_ primer sequences and PCR product lengths were as follows:

Nes: TTGCTTGTGGCCCTGAAAAG, CCAGCTGTGGCAGATGGATT, 129 bp

Sox2: CGCCGAGTGGAAACTTTTGT, CGCGGCCGGTATTTATAATC, 111 bp

Dcx: AAAGCTTCCCCAACACCTCA, CCATTTGCGTCTTGGTCGTTA, 101 bp

Fgfr1: AAATTCAAATGCCCGTCG, GGCGTAACGAACCTTGTAGCC, 91 bp

Egfr: CCCCACCACGTACCAGATG, GACACACGAGCCGTGATCTGT, 112 bp

Cxcl12 (Sdf1): atcagtgacggtaagccagtca, tgcttttcagccttgcaaca, 145 bp

Cxcr4: cgagcattgccatggaaatat, attgcccactatgccagtcaa, 170 bp

Actb: GGCCAACCGTGAAAAGATGA, GCCTGGATGGCTACGTACATG, 75 bp.

Probe hydrolysis assay for Vim was Rn00579738_m1 (Taqman, Applied Biosystems). The probe signal was normalized to an internal reference and a cycle threshold (Ct) was taken significantly above the background fluorescence. The Ct value used for subsequent calculation was the average of three replicates. The relative expression level was calculated using transcript level of Actb as endogenous reference. Data analysis was done according to the comparative method following the User Bulletin No. 2 (Applied Biosystems).

### Western blot analysis

Samples were isolated from rat meninges at different developmental stages (E20, P0, P15, and 6–8 weeks adult). Tissue was homogenized in PBS extraction solution with protease inhibitors and extracts were clarified by centrifugation. Protein concentration was determined by using the Bradford protein assay (Sigma). Protein content equivalent to 7 and 10 μg was diluted in loading buffer (Tris-HCl pH 6.8 12 mM, glycerol 20%, SDS 6%, β-mercaptoethanol 28.8 mM, EDTA 4 mM, bromophenol blue 0.2%) and loaded onto constant gradient polyacrylamide gel (10%). Proteins were separated by SDS-PAGE using Biorad electrophoresis system in running buffer (Tris 25 mM, glycine 19.2 mM, SDS 10%), with constant voltage set at 80 V for the entire electrophoresis run. Proteins were transferred onto PVDF membrane, previously equilibrated in methanol, at 60 V in transfer buffer (Tris 25 mM, glycine 19.2 mM, methanol 20%) under refrigerated conditions for 2 h using the Biorad electrophoresis system. Membranes were blocked with 5% BSA and 0.1% Tween-20 in Tris-buffered saline (TBS, pH 7.4) for 1 h and incubated overnight at 4°C with antibodies to DCX and β-actin, diluted in antibody solution (2.5% BSA and 0.1% Tween-20 in TBS pH 7.4): polyclonal rabbit-anti DCX (Cell Signaling; 1:750) (Dellarole and Grilli, 2008) and monoclonal mouse-anti β-actin (Sigma; 1:3000). After washing, membranes were incubated with appropriate HRP secondary antibody diluted in antibody solution for 1 h at room temperature; secondary antibody dilutions were: anti-rabbit IgG HRP conjugated (Chemicon) 1:5000 and anti-mouse IgG HRP (Millipore) 1:2000. Membranes were developed with a chemoluminescence system (ECL Plus, GE Healtcare) and proteins visualized on Hyperfilms (GE Healtcare). Autoradiographs were scanned by Kyocera scanner system.

### Transmission electron microscopy

For ultrastructure examination, brains from perfused rats were further fixed with 1% glutaraldeheyde in 0.1 M cacodylate buffer pH 7.2 for 30 min, sliced with razor blades, postfixed with 1% OsO4, dehydrated and embedded in Epon (Epon, Electron Microscopy Sciences, USA). Ultrathin sections were with a Philps CM10 transmission electron microscope.

### Statistical analysis

Data were analyzed using GraphPad Prism5 software. Differences between experimental conditions were analyzed using One-Way ANOVA with Tukey *post-hoc* test correction. *P* < 0.05 was considered statistically significant.

## Results

### Leptomeningeal cells and their proliferation during development

**Figure 1:**
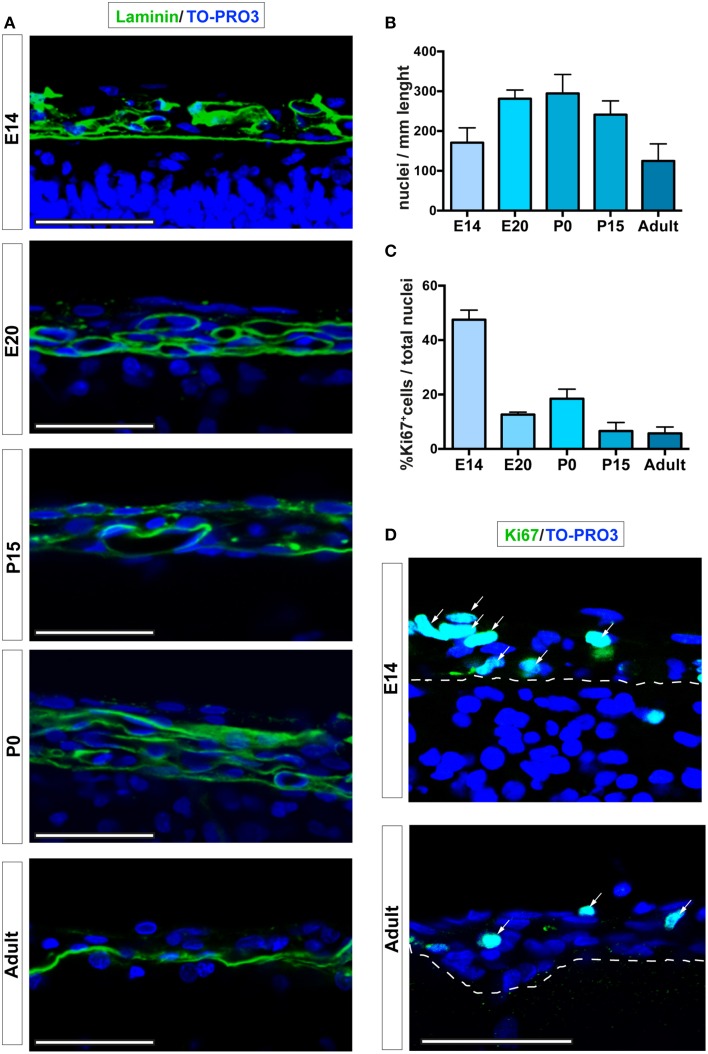
**Leptomeningeal cells at different developmental stages. (A)** Confocal microscopy analysis of leptomeninges at different stages of development; from top to bottom: E14, E20, P0, P15, adult. Basal lamina of the pia mater was visualized by laminin immunoreactivity (green). **(B)** Quantification of number of meningeal cell nuclei present along 1 mm of brain sections; number of nuclei peaked at P0. **(C)** Percentage of meningeal cells positive for the proliferation marker Ki67. The number of Ki67^+^ cells is maximum at E14 and decreases going to adulthood. The number of rats analyzed in **(B,C)** was *n* = 3 at E14, *n* = 6 at E20, *n* = 3 at P0, *n* = 5 at P15, and *n* = 4 at adulthood; values represent mean ± SD **(D)**. Confocal microscopy representative images of Ki67^+^ cells (green) of E14 and 6–8 weeks adult rat brain leptomeninges. Arrows indicate Ki67^+^ cells, the white dashed line highlights the border between neural parenchyma and meninges. Nuclei are stained with TO-PRO3 (blue). Scale bar: 50 μm.

To analyze the number and the proliferation of cells in the dorsal brain leptomeninges, we studied coronal sections obtained from embryonic (E14, E20), postnatal day 0 (P0) and 15 (P15) and adult (6–8 weeks) rats. The skull and the dura mater were removed from E20 onwards, whereas at E14 the coronal sections included the undifferentiated mesenchymal network of cells from which both the skull and the meninges will be formed. We used laminin, a component of the basal membrane, to visualize the pia mater and spatially distinguish between parenchymal and meningeal nuclei (Figure 1A). Immunofluorescence quantitative confocal analysis showed a Gaussian distribution of the number of cells in leptomeninges during the developmental stages, reaching a peak at P0 (170.8 ± 37.2; 281.3 ± 21.9; 294.6 ± 47.7; 241.1 ± 34.7; 125.0 ± 42.8 nuclei/mm at E14, E20, P0, P15, and 6–8 weeks adult respectively; Figures 1A,B).

To further characterize the meningeal tissue, we determined differences in cell proliferation, as defined by expression of the proliferation marker Ki67 (Bullwinkel et al., 2006). The highest fraction of proliferating leptomeningeal cells was observed at E14 (45.7% ± 2.5 of total nuclei, *n* = 3; Figures 1C,D). Although the value decreased with time, the percentage of proliferating cells remained relatively high at all developmental stages as well as in postnatal brains up to 8 weeks (Ki67-positive nuclei: 15.9% ± 2.2; 17.3% ± 7.3; 9.5% ± 9.4; 7.3% ± 6.2 of total nuclei at E20, P0, P15, and 6–8 weeks adult respectively; Figures 1C,D). Since distinction of the leptomeninges from dura mater and brain parenchyma is difficult and uncertain before E20, further assessments of the stem cell niche features of the leptomeninges were done starting from this embryonic day.

### Leptomeningeal cells express neural progenitor markers

**Figure 2:**
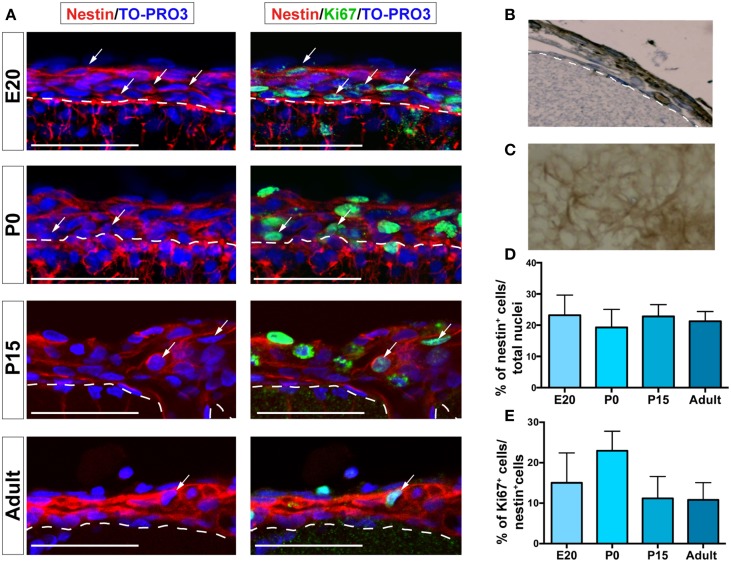
**Nestin^+^ and Ki67^+^ cells are present in leptomeninges. (A)** Immunostaining of nestin (red, left column) and nestin (red)/Ki67 (green, right column) at different stages of development; from top to bottom: E20, P0, P15, adult. Confocal microscopy analysis revealed that nestin^+^ cells (red) are present in the leptomeninges from embryonic stage E20 up to adulthood. Nuclei are stained with TO-PRO3 (blue). Scale bar: 50 μm. **(B,C)** Immunoperoxidase staining (brown) with anti-nestin antibody of brain sections. **(B)** Coronal section; the white dashed line highlights the border between neural parenchyma and meninges. **(C)** *En face* view showing nestin^+^ cells as an intricate net covering the brain. **(D)** Quantification of nestin^+^ cells normalized for the total number of nuclei in meninges. **(E)** Percentage of nestin^+^/Ki67^+^ cells; the values are normalized for the number of nestin^+^ cells. In **(D,E)**, values are mean ± SD.

The expression of the neural progenitor marker nestin (Decimo et al., 2012b) was analyzed by immunofluorescence confocal microscopy. Nestin is an intermediate filament expressed in all neural precursors and absent in differentiated neural cells (Lendahl et al., 1990). Although the absolute number of nestin-expressing cells in the leptomeninges (identified by their localization above the laminin-reactive pia mater) decreased constantly with age, their proportion remained constant (range from 19.3% ± 5.8 to 23.2% ± 6.5 of total meningeal cells) throughout the analyzed stages (Figures 2A,D, Table 1). As shown in Figures 2B,C, the distribution of nestin-expressing cells appeared as an intricate net of cells adjacent to the basal lamina. The fraction of nestin-positive cells that was also positive for the proliferation marker Ki67 was 15.0% ± 7.4 at E20, peaked at P0 (22.9% ± 10.8) and remained constant later on (11.2% ± 5.4 at P15 and 10.8% ± 4.3 at 6–8 weeks) (Figures 2A,E).

**Table 1:** **Quantitative analysis of neural precursor markers in leptomeninges at different developmental stages**.

	**Vimentin^+^ cells**	**Nestin^+^ cells**	**Vimentin^+^/Nestin^+^ cells**	**DCX^+^ cells**	**Sox2^+^ cells**
E20	25.7% ± 4.8 (*n* = 4)	23.2% ± 6.5 (*n* = 4)	12.9% ± 5.1 (*n* = 3)	10.5% ± 4.4 (*n* = 4)	2.6% ± 1.1 (*n* = 3)
P0	20.6% ± 3.9 (*n* = 4)	19.3% ± 5.8 (*n* = 4)	8.2% ± 1.3 (*n* = 4)	13.0% ± 4.1 (*n* = 4)	4.2% ± 3.4 (*n* = 3)
P15	24.8% ± 4.5 (*n* = 5)	22.8% ± 3.8 (*n* = 3)	7.8% ± 1.9 (*n* = 3)	4.8% ± 5.2 (*n* = 4)	0.2% ± 0.3 (*n* = 3)
Young adult (6–8 weeks)	31.0% ± 0.8 (*n* = 3)	21.3% ± 3.1 (*n* = 3)	8.2% ± 1.8 (*n* = 3)	1.4% ± 1.1 (*n* = 3)	0.5% ± 0.5 (*n* = 3)
Mature adult (24 weeks)	32.2% ± 5.4 (*n* = 3)	17.7% ± 1.4 (*n* = 3)	5.5% ± 3.1 (*n* = 3)	1.4% ± 0.7 (*n* = 3)	0.3% ± 0.6 (*n* = 3)

*Quantifications were performed by counting cells lying above the pial basal lamina (visualized by laminin immunoreactivity). Numbers are expressed as the percentage of positive cells on the total number of cells counted. Data are mean ± SD; n = number of animals analyzed*.

**Figure 3:**
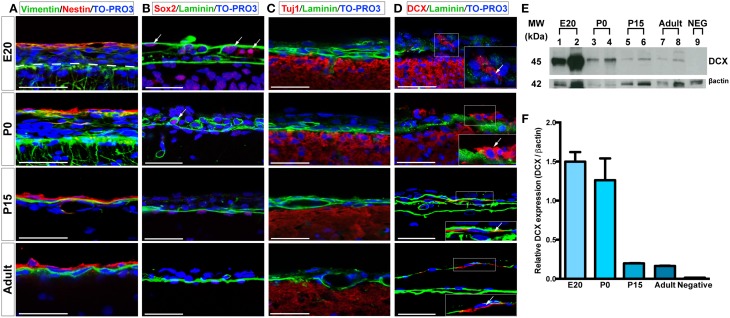
**Expression of neural progenitors markers in the leptomeninges. (A–D)** Confocal microscopy images of leptomeninges in brain coronal sections stained with vimentin (green)/nestin (red) (A), Sox2 (red)/laminin (green) **(B)**, Tuj1 (red)/laminin (green) **(C)**, and DCX (red)/laminin (green) **(D)** at different stages of development; from top to bottom: E20, P0, P15, adult. Arrows in **(B,D)** point to Sox2^+^ and DCX^+^ cells respectively. Nuclei are stained with TO-PRO3 (blue). Scale bar: 50 μm. **(E)** Western Blot of meninges lysates. 7 μg of total protein lysate were loaded in lanes 1, 3, 5, 7, 9 and 10 μg in lanes 2, 4, 6, 8. Lanes 1–2: lysates from E20 meninges. Lanes 3–4: lysates from P0 meninges. Lanes 5–6: lysates from P15 meninges. Lanes 7–8: lysates from adult meninges. Lane 9: lysates from P0 meninges as negative control for the secondary antibody. Numbers on the left indicate molecular masses in kilodaltons (kDa). **(F)** Densitometric analysis of relative protein levels shown in **(E)**. DCX expression was normalized for β-actin expression. DCX relative expression is high in E20 and P0 meningeal lysates and persists in P15 and adult meningeal lysates.

We further assessed the presence and distribution of additional neural progenitor markers in the leptomeninges, including vimentin (Stagaard and Mollgard, 1989), Sox2 (Zappone et al., 2000), doublecortin (DCX) (Dellarole and Grilli, 2008), and βIII Tubulin (Tuj1) (Caccamo et al., 1989); for these markers, analysis was extended to 24 weeks-old rats. Vimentin, a type III intermediate filament protein expressed in neural stem cells as well as in mesenchymal cells (Stagaard and Mollgard, 1989; Decimo et al., 2012b), was present in leptomeningeal cells at all stages (Figure 3A, Table 1). At E20, we observed vimentin- and nestin-double positive cells, while starting from P0, a distinct layer of nestin-positive/vimentin-negative cells appeared. From P15 to adult, nestin-positive and vimentin-positive cells formed distinct layers, however, a fraction of vimentin- /nestin-double positive cells persisted (Table 1).

The transcription factor Sox2 is expressed in the neural tube throughout development as well as in postnatal neural progenitors (Zappone et al., 2000). Interestingly, we detected Sox2 immunoreactivity in all the analyzed time points, with higher percentages in embryonic and early postnatal days (Figure 3B, Table 1). In the adult, Sox2-expressing cells in the meninges were extremely rare, whereas they were located in the brain parenchyma underneath the pia mater basal lamina (Figure 3B, Table 1).

We also assessed the distribution of neural progenitor markers that have been shown to be expressed at later stages of neuronal precursor differentiation, such as Tuj1 and DCX (Caccamo et al., 1989; Dellarole and Grilli, 2008). No Tuj1-expressing cells were observed in meninges (Figure 3C); on the contrary, a limited number of leptomeningeal cells expressed DCX during development up to adult stages (Figure 3D, Table 1). The presence of DCX protein in meninges at all the developmental stages was confirmed by western blot (WB) analysis: as expected, the amount of DCX present in meninges decreased with age but was still detectable in adult brains (Figures 3E,F).

**Figure 4:**
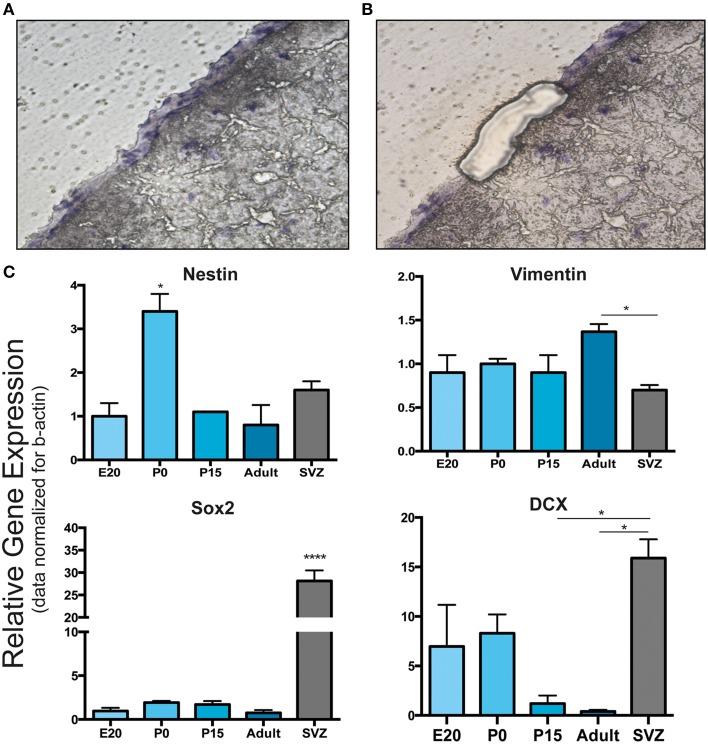
**Laser capture microdissection and gene expression analysis of leptomeningeal cells. (A,B)** Laser capture microdissection (LCM) was performed to distinguish leptomeningeal from parenchymal gene expression. **(A)** Shows a coronal brain section with the entire meningeal layer before LCM. **(B)** Shows the same section after meningeal dissection. From each stage of development (E20, P0, P15, adult), at least 1000 cells were collected from meningeal tissue **(B)**. **(C)** qRT-PCR on collected samples was performed for gene expression analysis. As expected from immunofluorescence and WB analysis, we detected expression of nestin, vimentin, Sox2 and DCX genes. Expression of all these neural precursor-related genes persisted up to adulthood. SVZ samples from 6 to 8 weeks adult rats were used as positive control. ^*^p < 0.05; ^****^p < 0.0001. Values are mean ± SEM of 3 replicates.

The presence of these neural precursor markers in meninges was further analyzed at the gene expression level. To clearly distinguish leptomeningeal from parenchymal gene expression, we performed laser capture microdissection (LCM) of meningeal tissue and carried out qRT-PCR on the collected samples for gene expression analysis (Figures 4A,B); SVZ tissue isolated from 6 to 8 weeks adult rats was used as positive control. Consistently with the immunofluorescence and WB analysis, we detected expression of nestin, vimentin, Sox2 and DCX genes at all stages including adulthood (Figure 4C). We observed that leptomeningeal gene expression levels of nestin and vimentin genes were comparable to SVZ, while Sox2 and DCX genes were expressed at lower levels, suggesting differences in cellular composition between the two tissues.

These results suggest that leptomeninges host precursor cells expressing nestin, vimentin, Sox2 and DCX during development. Nestin expressing meningeal cells appeared to be abundant and to retain proliferation properties from embryo until adulthood.

### Major extracellular components of the meningeal tissue during development

**Figure 5:**
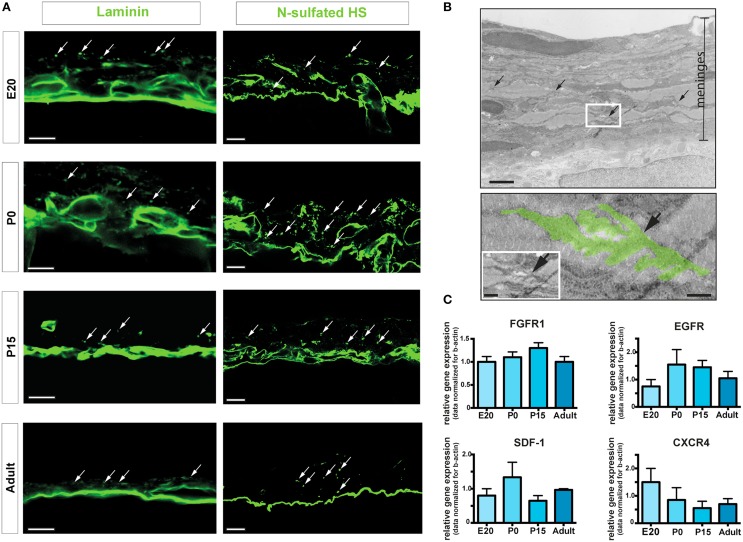
**ECM components and fractones. (A)** Confocal images of meninges in brain coronal sections showing the presence of immunoreactivities for laminin and N-sulfated HS. Dot-like aggregates (arrows) suggest organization of fractones in the leptomeninges. Scale bar: 10 μm. **(B)** Transmission electron microscopy representative image of P15 rat meninges. The white rectangle in the upper picture is enlarged in the lower frame; colored area highlights a fractone. Scale bar: 5 μm upper panel; 0.5 μm bottom panel and 1 μm in the insert. **(C)** Relative gene expression of FGFR1, EGFR, SDF1, and CXCR4 of rat leptomeninges at E20, P0, P15, and 6–8 weeks adult. Values are mean ± SEM of 3 replicates.

Neural stem cell niches are characterized by the presence of extracellular matrix components and chemotactic factors (Kerever et al., 2007; Kokovay et al., 2010). Accordingly, we assessed the presence of laminin and N-sulfated heparan sulfate (N-sulfated HS), a member of the glycosaminoglycan family that has been shown to bind and concentrate growth factors, including FGF2 and epidermal growth factor (EGF) (Yayon et al., 1991; Mercier and Arikawa-Hirasawa, 2012). Immunoreactivities for laminin and N-sulfated HS were observed by confocal microscopy in brain leptomeninges at all the developmental stages analyzed (Figure 5A). Interestingly, both laminin and N-sulfated HS were present in vascular basement membranes and in fractones (Mercier et al., 2002), specialized extracellular matrix structures appearing as series of immunoreactive puncta aligned along the meninges (arrows in Figure 5A). Fractones were also observed at the ultrastructural level (Figure 5B), where they appeared as electrondense material formed by extravascular basal lamina with typical folds and tube-like morphology and measuring 5–10 μm in length and 1–4 μm in diameter (Figure 5B). Meningeal fractones were similar to fractones described in the SVZ (Mercier et al., 2002), suggesting that meninges are endowed, during development and in adulthood as well, with extracellular matrix organized in specific structures that promote heparin-binding growth factor activity and cell proliferation. Indeed, growth factors relevant for neural development, such as FGF2 and heparin binding-EGF, have been found in meninges (Nakagawa et al., 1998; Mercier and Hatton, 2001).

In line with these findings, we detected gene expression of the growth factor receptors FGFR1 and EGFR in leptomeninges at all-time points of analysis (Figure 5C). Moreover, the chemotactic factor SDF-1 and its receptor CXC chemokine receptor 4 (CXCR4) were also expressed in leptomeninges at all the developmental stages analyzed (Figure 5C). SDF-1 and its receptor CXCR4 are known to be involved in homing, movement, proliferation and differentiation of progenitor cells (Kokovay et al., 2010), further indicating that leptomeninges may be a niche for neural progenitors.

Collectively, these data suggest that the extracellular components of the meninges form a microenvironment favoring homing and proliferation of precursor cells.

## Discussion

Previous works described the presence in adult meninges of a stem cell-like population that reacts to CNS injury by displaying the hallmarks of a neural stem cell niche: activation, increased proliferation and migration to the lesioned parenchyma (Decimo et al., 2011; Nakagomi et al., 2011, 2012; Ninomiya et al., 2013; Kumar et al., 2014). Moreover, a population of nestin-positive cells could be extracted from meningeal tissue, cultured *in vitro* and showed neural differentiation potential *in vitro* and after transplantation *in vivo* (Bifari et al., 2009; Nakagomi et al., 2011). These observations led us to further investigate whether meninges possess the features described for canonical neural stem cell niches (Bjornsson et al., 2015) and whether these features also persist at the end of the developmental period.

### Cell expressing neural precursor markers are retained in meninges

The neural stem cell niche is a tissue microenvironment capable of hosting and maintaining neural progenitor cells for the lifetime (Scadden, 2006; Decimo et al., 2012b). It ensures a unique microenvironment where interactions between cells, extracellular matrix molecules (ECM) and soluble signals, provide the proper control of neural precursor renewal and differentiation (Scadden, 2006; Decimo et al., 2012a,b; Bjornsson et al., 2015).

All these features are expressed and maintained in adulthood in the most studied neurogenic niches, i.e., the subventricular zone (SVZ). At this site, different cell types are present, including quiescent NSCs, transient amplifying precursors and committed neuroblasts, each expressing specific sets of markers (Doetsch et al., 1997). With this study we show that leptomeninges harbor a population of cells expressing the undifferentiated neural precursor markers nestin, vimentin and Sox2. Approximately 20% of the leptomeningeal cells expressed nestin and roughly 15% of those cells were in the active phase of the cell cycle in all the stages analyzed. At all time-points, a small fraction of meningeal cells also expressed DCX, a microtubule-associated protein expressed by neuronal precursor cells and immature neurons in embryonic and adult cortical structures. Thus, similar to the SVZ, leptomeninges host a subset of cells expressing markers of undifferentiated, proliferating and differentiating neural precursors and this set of cells persists in adulthood. Thus, meninges may represent a functional niche for progenitors during embryonic development and in adulthood.

Although leptomeninges share several features of the SVZ niche, our data also highlight quantitative differences in Sox2 and DCX gene expression levels between these two tissues, possibly reflecting differences in cell composition and in functional significance for brain homeostasis.

### Leptomeninges possess molecules necessary to form a microenvironment favoring proliferation and homing of precursor cells

In SVZ distinct ECM components and chemotactic factors have been described, including FGF2 and epidermal growth factor (EGF) (Yayon et al., 1991; Mercier and Arikawa-Hirasawa, 2012), as well as components of chemoattractant signaling systems such as SDF-1 and its receptor CXCR4. Members of this signaling machinery act in concert, as shown by SDF-1-induced stimulation in EGFR-expressing cells of movement toward the blood vessel surface, proliferation and generation of transient amplifying cells (Kokovay et al., 2010).

Our gene expression data confirm that similar signaling machinery is present in meninges. Indeed, we found expression of the growth factor receptors FGFR1 and EGFR in leptomeninges, as well as of the homing chemotactic factor SDF-1 and its receptor CXCR4 from embryonic to adult stages. Our data are in line with published results showing that meninges are highly responsive to several mitogens, including EGF, FGF-2 and BDNF (Day-Lollini et al., 1997; Parr and Tator, 2007). Moreover, SDF-1 secreted by meningeal cells acts as chemotactic factor on neural cells (Borrell and Marin, 2006). Interestingly, modulation of this chemoattractant signaling system was observed following spinal cord injury (increase of CXCR4/SDF-1 ratio) (Decimo et al., 2011).

The persistent expression in meninges of these important signals for proliferation, homing and migration of neural progenitors suggests that cellular dynamics in the CNS are complex and that, depending on the needs of the brain parenchyma, the meningeal niche may adapt its signals promoting either proliferation, migration or homing. In this context, it is important to note that our data indicate the presence of fractones at all stages of life, including both development and adulthood. Fractones are specialized extracellular matrix structures that appear to bind and concentrate important regulators of proliferation and migration (Kerever et al., 2007; Mercier and Arikawa-Hirasawa, 2012). These N-sulfated HS structures have been described in detail both in rodent and human brains: they are present associated to well-described sites of adult neurogenesis such as the SVZ and the hippocampus and appear to form a continuum across these neurogenic niches connecting them to the olfactory bulb, the rostral migratory stream, the sub-callosum, the subcapsule zones and the meninges (Mercier and Arikawa-Hirasawa, 2012). This confirms that meninges have the potential to connect different portions of the brain.

In line with the idea that meninges play a pivotal role in guiding stem cells migration in the brain, are our observations that transplanted leptomeningeal stem cells accumulate in meninges following injection in the third ventricle of adult animals [unpublished observations] and the finding of ectopic colonies at the pial surface of the spinal cord following embryonic neural stem cells transplantation at the site of injury (Steward et al., 2014).

## Conclusion

This study provides a new and accurate description of the molecular and cellular aspects of meninges related to their newly identified function of niche for neural progenitor/stem cells. We add to previous information the notion that this niche is indeed present and potentially active at all stages of development and in adult life as well. The identification of receptors for trophic factors, of ECM components and chemotactic factors known to be involved in homing, movement, proliferation and differentiation of progenitor cells strengthens the idea that the niche function of meninges is not limited to conditions associated to diseases, such as injury or ischemia (Decimo et al., 2011; Nakagomi et al., 2012).

Our description of the molecular and cellular properties of the meningeal niche in healthy animals calls for a physiological function of this progenitor niche. The notion that neurogenesis may occur in response to physiological and not just pathological stimuli is well accepted (Kempermann et al., 1997); although the earliest and the most abundant information have been obtained from well identified structures including SVZ and hippocampus, data indicate that neurogenesis may also occur in response to physiological stimuli at sites that are distant from those classical niches (Dayer et al., 2005). In this context, we propose that meninges may be a wide-spread niche from where neurogenesis may be induced on demands following physiological stimuli; alternatively, or in addition, meninges may serve as a highway for delivery to distant sites of neural precursors newly generated in classical neurogenic niches. Further studies tracing the fate of meningeal cells are therefore needed to clarify the functional significance of this newly discovered niche and to determine the potential role of meninges in brain homeostasis.

## Author contribution

All authors performed research and/or analyzed data; FB, VB, GF, and ID designed research and wrote the paper. All authors discussed the results and commented on the manuscript.

### Conflict of interest statement

The authors declare that the research was conducted in the absence of any commercial or financial relationships that could be construed as a potential conflict of interest.
